# Bone-Marrow Stem Cells and Acellular Human Amniotic Membrane in a Rat Model of Heart Failure

**DOI:** 10.3390/life11090958

**Published:** 2021-09-13

**Authors:** Gustavo Gavazzoni Blume, Paulo André Bispo Machado-Junior, Rossana Baggio Simeoni, Giovana Paludo Bertinato, Murilo Sgarbossa Tonial, Seigo Nagashima, Ricardo Aurino Pinho, Lucia de Noronha, Marcia Olandoski, Katherine Athayde Teixeira de Carvalho, Julio Cesar Francisco, Luiz Cesar Guarita-Souza

**Affiliations:** 1 Experimental Laboratory of Institute of Biological and Health Sciences, Pontifícia Universidade Católica do Paraná (PUCPR), Curitiba 80215-901, Brazil; machadojrpab@gmail.com (P.A.B.M.-J.); rossanabaggio@gmail.com (R.B.S.); gigibertinato@gmail.com (G.P.B.); murilotonial@gmail.com (M.S.T.); seigocap@gmail.com (S.N.); lnno.noronha@gmail.com (L.d.N.); bio.estatistica@pucpr.br (M.O.); guaritasouzalc@hotmail.com (L.C.G.-S.); 2 Laboratory of Exercise Biochemistry in Health, Graduate Program in Health Sciences, School of Medicine, Pontifícia Universidade Católica do Paraná, Curitiba 80215-901, Brazil; rapinho12@gmail.com; 3 Advanced Therapy and Cellular Biotechnology in Regenerative Medicine Department, The Pelé Pequeno Príncipe Institute, Child and Adolescent Health Research & Pequeno Príncipe Faculties, Curitiba 80215-901, Brazil; katherinecarv@gmail.com (K.A.T.d.C.); julio.apfr@gmail.com (J.C.F.)

**Keywords:** myocardial infarction, tissue regeneration, stem cells, human amniotic membrane

## Abstract

Myocardial infarction (MI) remains the leading cause of cardiovascular death worldwide and a major cause of heart failure. Recent studies have suggested that cell-based therapies with bone marrow stem cells (BMSC) and human amniotic membrane (hAM) would recover the ventricular function after MI; however, the mechanisms underlying these effects are still controversial. Herein, we aimed to compare the effects of BMSC and hAM in a rat model of heart failure. MI was induced through coronary occlusion, and animals with an ejection fraction (EF) < 50% were included and randomized into three groups: control, BMSC, and hAM. The BMSC and hAM groups were implanted on the anterior ventricular wall seven days after MI, and a new echocardiographic analysis was performed on the 30th day, followed by euthanasia. The echocardiographic results after 30 days showed significant improvements on EF and left-ventricular end-sistolic and end-diastolic volumes in both BMSC and hAM groups, without significant benefits in the control group. New blood vessels, desmine-positive cells and connexin-43 expression were also elevated in both BMSC and hAM groups. These results suggest a recovery of global cardiac function with the therapeutic use of both BMSC and hAM, associated with angiogenesis and cardiomyocyte regeneration after 30 days.

## 1. Introduction

Acute myocardial infarction (AMI) is the most common cause of death globally, accounting for 32% of deaths worldwide in 2013 [1]. The majority of the events are related to a fissure and consequent rupture of atherosclerotic plaque, with consequent thrombus formation and obstruction of the coronary artery [2].

In patients who survive, significant modifications occur in the electrical, biochemical, and structural architecture of the ventricular wall, mainly related to the action of pro-inflammatory cytokines, macrophages, and neutrophils in the infarct area [3,4]. The cellular hypoxia leads to oxidative stress and formation of free radicals, which are able to damage crucial proteins necessary for myocardial excitation and contraction, such as ion channels and calcium pumps, with consequent loss of contractile function, resulting in cardiac remodeling and ventricular dysfunction [3,5,6].

The ventricular dysfunction is a major cause of heart failure (HF). Data from the American Heart Association estimated a prevalence of 5.1 million individuals with HF in the United States in 2012, with projections of a 46% increase by 2030, resulting in more than 8 million people being affected by this complication [7,8,9]. HF is also the main cause of hospitalization worldwide in individuals older than 65 years, generating an annual cost of 26 billion USD in the United States [7].

These numbers are mostly related to the limitations of the current therapeutic approaches after coronary occlusion, which are able to treat only the consequences of AMI, not being able to restore the contractility of the necrotic cells. Thus, the evolution to HF is inevitable in most of the cases, which turns heart transplantation as the last effective therapy for these patients. This procedure, even though it is considered as the gold-standard treatment for HF [10], is not always an option due to the scarcity of donors and comorbidities that usually contraindicate the transplant [10].

In this scenario, new therapeutic options, such as cell-based therapies, emerge as an alternative for the treatment of HF, due to the potential of restoring cardiac function, reducing infarct size and preventing cardiac remodeling [3,11]. Previous animal studies have demonstrated improvements in ventricular function with the use of bone marrow stem cells (BMSC) after AMI, showing the potential of angiogenesis, and the formation of undifferentiated cells and new cardiomyocytes [12,13]. Despite this, its ability to reverse ventricular remodeling is still controversial in the literature [14,15].

On the other hand, the human amniotic membrane (hAM), previously used in skin wounds, ophthalmological and gynecological conditions, has been shown to prevent cardiac remodeling in animals submitted to ischemic events [16,17,18] with anti-inflammatory and antimicrobial properties [19,20,21]. Moreover, it is a matrix that does not trigger an immune response in the host on its decellularized form, making it even more attractive for grafting models in tissue engineering [21,22].

Despite this, the mechanisms involving the action of both hAM and BMSC and the interaction with the resident cardiac cells are still not well-established in the literature. In this way, understanding the histopathological processes that occur in the presence of these materials after AMI is highly relevant in choosing an effective therapeutic approach. In this sense, the present study aimed to evaluate the functional and histopathological effects of BMSC and hAM in a rat model of myocardial infarction.

## 2. Materials and Methods

This is a randomized animal experimental study. The experiments were performed following the ethical standards and principles of the Brazilian College of Animal Experimentation—COBEA—with approval from the Research Ethics Committee on the use of animals at PUCPR—CEUA/PUCPR, under approval protocol 1273/2018.

Briefly, a total of 50 male Wistar rats, 2–3 months old, were submitted to AMI. After proof by echocardiography of infarction after 7 days, the animals were randomized and divided into 3 groups: Control; Human Amniotic Membrane (hAM); Bone-marrow Mononuclear Stem Cells (BMSC). On the 30th day after myocardial infarction, the animals were submitted to a new echocardiographic evaluation and subsequently euthanized, followed by histopathological analysis of the transmural infarction in the compromised region.

### 2.1. Animals

For the study, 50 adult male Wistar rats (*Rattus norvegicus albinus*, *Rodentia mammalia*) weighing between 250 and 300 grams were used. The animals were housed in cages and kept under constant environmental conditions, with a 12-h day/night cycle, room temperature, and water and feed for free consumption.

### 2.2. Myocardial Infarction Induction

The animals were submitted to intramuscular general anesthesia, with administration of Ketamine (Ketamin^®^/Cristalia–50 mg/kg) and Xylazine (Calmiun^®^/Agener União–10 mg/kg). After reaching the anesthetic plane, verified through the eyelid reflex and muscle relaxation, a definitive airway was obtained through orotracheal intubation with a number 14 peripheral venous catheter. After that, the animals were connected to a mechanical ventilation system with a volume of 2.5 mL (O_2_/min) and a frequency of 50 cycles per minute, observing lung expansion. Two volume respirators were used (HARVARD^®^, Inc., respirator model 683, Holliston, MA, USA) for small animals with oxygen at 21% (room air).

With the rat anesthetized and ventilated, the animal was positioned in dorsal decubitus with a slight inclination to the right, facilitating the exposure of the area. The forelimbs and hind limbs were fixed with adhesive tape. After adequate positioning, antisepsis of the thorax was performed with topical iodopovedine, and a left lateral thoracotomy was performed on the third intercostal space. After opening the left pleura, the pericardium was opened for dislocation and better visualization of the area to be approached. Followed by exposure of the heart, the left auricle was retracted, and the left coronary artery located between the pulmonary artery and the left atrium. The ligature of the coronary artery was performed with non-absorbable 7-0 monofilament polypropylene suture thread [23]. The infarcted area was immediately visualized by the difference in coloration of the affected area. After that, the heart was repositioned to the thorax, the lungs were hyperinflated, and the chest wall was closed by planes with 4-0 non-absorbable monofilament mono-nylon suture thread. For postoperative analgesia, non-steroidal anti-inflammatory drug (Carprofen), sterile injectable solution 2–5 mg/mL was used.

### 2.3. Echocardiography (7th Day)

The animals were submitted to echocardiographic analysis using two-dimensional echocardiography equipment model HD7 (Philips Medical Systems, Andover, MA, USA), with S12 sectorial (5–12 mHz) transducer.

All animals, regardless of the group, were submitted to an echocardiographic evaluation on the seventh day after coronary occlusion and anesthetized with ketamine and xylazine at a dose of 25 mg/kg and 5 mg/kg, respectively, intramuscularly. All measurements were made blindly and three times by the same observer, and the final result was an average of the three measurements. The main objective of the echocardiogram was to evaluate the Ejection Fraction (EF) and end-systolic and end-diastolic volumes of the left ventricle of the animals. Those with EF > 50% were excluded from the study.

### 2.4. Preparation of Human Amniotic Membrane (hAM)

The amniotic membranes were obtained from parturient women (n = 2, at 36 and 40 weeks of gestational age), after signing the Free and Informed Consent form according to protocol approved by the ethics committee for research on human beings of the Hospital Pequeno Príncipe—protocol number 659.204/2014 and 0948-11.

The amniotic membranes were obtained after cesarean deliveries. Maternal donors were serologically negative for HIV, hepatitis B, hepatitis C, and syphilis. Possible blood clots were immediately removed from the placenta with phosphate-buffered saline solution (PBS) containing 100 u/mL penicillin and 100 mg/mL streptomycin. Amniotic epithelial cells were removed from the human amniotic membrane using SDS (sodium duodecyl sulfate) solution in PBS and incubated with a rotation speed of 100 rpm at 37 °C for 24 h, and finally, it was washed 3 more times with PBS.

Decellularization was performed aseptically in a BioSAFE class II biological safety cabinet (Veco^®^). For this process, the membranes were removed from the solution (PBS) buffer phosphate pH 7.2 (Gibco) and treated with 0.01% SDS and 0.01% SD (sodium deoxycholate) solution for 24 h at 37 °C with the aid of a mechanical shaker (Mesa Agitadora 109 M, Nova Ética Ltd., Lemesos, Cyprus). Then, the membranes were preserved in PBS at 4 °C, according to the methodology described in previous studies [24,25]. In order to guarantee the decellularization of the hAM, phase-contrast and fluorescence microscopic analyses of Hoechst 33,258 staining were performed, confirming the absence of cells in the sample.

### 2.5. Isolation of Bone Marrow Stem Cells (BMSC)

Bone marrow stem cells were obtained via bone-marrow aspiration of the iliac crest from the same animals that underwent surgical procedures, always preceded by anesthesia. The animals were placed in the lateral decubitus position with the upper leg flexed and the lower leg straight. The puncture aspiration was performed in the posterior iliac crest of the femur using a 5 mL disposable syringe (BD-Plastipak^®^) with 0.2 mL of heparin (5.000 IU/mL) with a 25 × 8 × 21 mm G1 needle (BD-Precision Glide^®^); about 1 mL of blood was collected from the bone marrow of each rat, followed by identification of the syringes.

The mononuclear fraction was obtained through density gradient (d = 1.077 g/m^3^) [26] in Iscove’s modified Dulbecco’s medium (DMEM-GIBCO BRL) supplemented with 1% antibiotic (penicillin and streptomycin) and 20% buffer solution. The material collected from each mouse was placed in a sterile 15 mL plastic centrifuge tube, and this tube was then completed to 12 mL with DMEM culture medium supplemented with 4% buffer solution and 1% antibiotic (penicillin and streptomycin) and homogenized. In a 15 mL plastic tube, 3 mL of density gradient separation solution (Ficoll-Hypaque) was placed with the homogenized content containing the animal’s bone marrow and DMEM culture medium. This tube was taken to the centrifuge and submitted to 1500 rpm rotations per minute for 40 min at 22 °C. After that, it was brought to flow again, and the ring formed between the medium and the gradient was removed. The stem cells were directly transplanted after isolation, and the transplant was considered autologous, with no need of immunosuppression for the animals. Our research group has established this expertise of bone marrow aspirate to obtain BMMC isolation in rats for preclinical research.

### 2.6. BMSC Transplantation and hAM Implantation

Animals with EF < 50% were randomly assigned into three groups 07 days after AMI: the control group, BMSC group, and hAM group. The animals were again anesthetized (50 mg/kg Ketamine and 10 mg/kg Xylazine) and subjected to median transsternal sternotomy. The BMSC group received 5 × 10^6^ mononuclear stem cells injected in multiple sites directly on the infarcted area and transition zone, as previously described [27], and the hAM group received a patch of hAM, measuring approximately 2 cm × 3 cm, on the anterior left ventricle surface; this patch was sutured with 7-0 polypropylene over the ischemic area identified under direct visualization. The control group received only saline solution by transsternal sternotomy.

### 2.7. Echocardiography (30th Day)

On the 30th day after coronary occlusion, all the animals were submitted to a new echocardiographic analysis, under the same conditions as described on the 7th day. The main objective was to compare EF, LVESV and LVEDV between pre- and post-application of hAM and BMSC periods. After the echocardiographic evaluation, euthanasia was performed with an overdose of the anesthetic drugs.

### 2.8. Anatomopathological Studies

All animals were euthanized, and their hearts were removed for histopathological analysis 30 days after implantation. The hearts were preserved in flasks containing 10% formaldehyde for 24 h. After this period, they were cleaved in four equal transversal parts in a microtome (Leica model RM 2145) with a thickness of 5 µm.

Once the sections were dehydrated, the pieces were submitted to successive baths in 70%, 80%, and 90% alcohol and three baths in 100% alcohol (Leica model TP1020) for one hour. Immediately thereafter, there was impregnation of liquid paraffin on the sections through three baths at 65 °C in the same device. Subsequently, the histological sections were mounted on slides and stained with H & E solution and Picro Sirus Red. From each fragment, two slides were made with the four sections and the mentioned stains.

#### 2.8.1. Collagen Deposition Assessment

The middle layer of the left ventricular sections (5 µm) was hydrated with Sirius Red (0.5% in saturated aqueous picric acid (Sigma, Kawasaki-shi, Japan) and viewed under polarized light. Ten fields in each region of the heart were randomly selected with three sections in non-consecutive series, and collagen content was quantified as Sirius Red positive areas by use of a Zeiss Axiovert S100 TV Microscope (Zeiss, Jena, Germany) 2.0 (UTHSCSA, San Antonio, TX, USA).

#### 2.8.2. Immunohistochemical Analysis

Histological slides were prepared with hematoxylin-eosin (HE) stain and were used to mark areas of interest for performing tissue sample array arrangement or Tissue Microarray (TMA). Next, two 4 µm-thick paraffin-embedded sections of the TMA blocks were transferred to electrically charged Star Frost™ (Braunschweig, Germany) slides and incubated with an anti-Desmine (ab8592; Abcam, Cambridge, UK), anti-connexin 43 (ab217676; Abcam, Cambridge, UK) and anti-factor VIII (275376; Abcam, Cambridge, UK) overnight in a humidified chamber at a temperature between 2–8 °C [28].

Immunoreactivity was developed by adding DAB chromogen-substrate solution (Spring) to the slides. Harris hematoxylin was used for counterstaining. Positive and negative controls were performed in parallel with all reactions. The slides were scanned using the Axio Scan.Z1 scanner (Carl Zeiss, Jena, Germany). The generated files were fragmented into single images and approximately 25 images were selected for analysis. The areas of immunopositive markings for the antiprotein antibodies were quantified using Image-Pro Plus version 4.5 software (Media Cybernetics, Rockville, MD, USA). The immunopositive objects were selected using a “mask” to standardize and automate the process. Numerical data of the immunopositive marking area were generated and subsequently exported to an Excel spreadsheet.

### 2.9. Statistical Analysis

Quantitative variables were described by means, medians, minimum values, maximum values, and standard deviation. For comparison of the groups, echocardiographic analysis at 07 days was considered the model of analysis of variance with one factor (ANOVA) and the test of covariance (ANCOVA) to evaluate after 30 days and compare with the results of baseline levels. The non-parametric Kruskal–Wallis test was used for the histopathological analyses. Values of *p* < 0.05 indicated statistical significance. The data were analyzed with the Statistica v.8.0 computer program.

## 3. Results

**Scheme 1:**
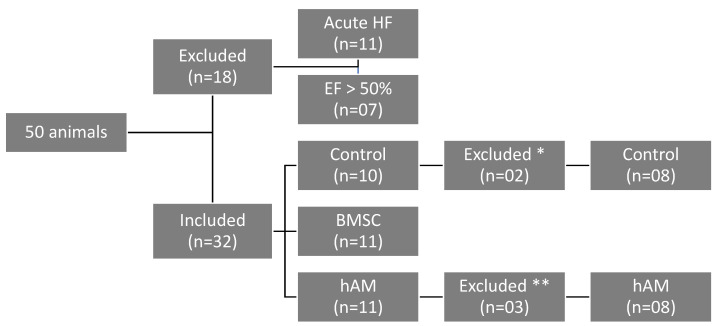
A total of 50 male rats were initially included in the study, but 18 animals were excluded after 07 days due to EF > 50% (07 animals) or death related to acute heart failure (n = 11); thus, a total of 32 animals were randomized in the control group (n = 10), BMSC (n = 11), and hAM (n = 11). After 30 days, 03 animals from the control group died immediately after saline injection procedure (*) and 03 animals died immediately after hAM implantation (**), leaving 27 animals that were included in the final analysis: control (n = 08), BMSC (n = 11), and hAM (n = 08).

Of the 50 animals initially included in the study, 11 died hours or days after infarction induction (mortality rate of 22%), and 07 were excluded for having an ejection fraction higher than 50%. Thus, before cell and membrane implantation procedures, 32 animals were included and randomized into three groups: control (n = 10), BMSC (n = 11) and hAM (n = 11). After applying the membranes, 03 animals of the hAM group and 02 animals of the control group died, related to postoperative complications, leaving 27 animals that were included in the final analysis: control (n = 08), BMSC (n = 11) and hAM (n = 08) (Scheme 1).

### 3.1. Echocardiographic Analysis

#### 3.1.1. Ejection Fraction (EF)

**Figure 1:**
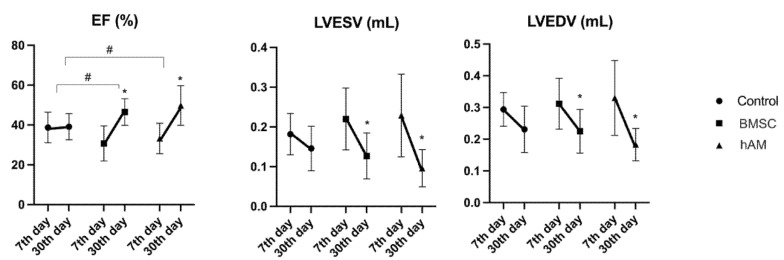
Echocardiographic results of the analyzed groups. Results are shown as mean ± standard deviation. *p* < 0.05 denoted statistical significance in comparison to control group (#) or intragroup seventh day analysis (*). *EF*: ejection fraction; *LVESV*: left ventricle end-systolic volume; *LVEDV*: left ventricle end-diastolic volume; *BMSC*: bone marrow mononuclear stem cells; *hAM*: human amniotic membrane.

The intergroup analysis showed no statistical difference (*p =* 0.122) between the groups on the seventh day after AMI, with values of 38.73% (control group), 33.21% (hAM group) and 30.67% (BMSC group); thus, the groups were homogeneous (Table 1, Figure 1). After 30 days, a significant difference was identified between the hAM and BMSC groups in comparison to the control group (*p* = 0.006 and *p* = 0.034, respectively). There was no significant difference in EF after 30 days when the hAM and BMSC groups were compared to each other (*p* = 0.326) (Table 2).

In the intragroup analysis after 30 days, the EF variated from 33.21% to 49.77% in the hAM group (*p* = 0.001) and from 30.67% to 46.49% in the BMSC (*p* < 0.001). No significant variation in EF was observed in the control group, (Table 1).

#### 3.1.2. Left Ventricular End-Systolic Volume (LVESV)

Regarding the LVESV, the intergroup analysis after 07 days showed values of 0.182 mL in the control group, 0.229 mL in hAM group and 0.220 mL in BMSC group, (*p* = 0.471). After 30 days, we identified values of 0.146 mL in the control group, 0.096 mL in the hAM group, and 0.127 mL in BMSC group (*p* = 0.056). Thus, the groups were considered homogeneous in both pre- and postoperative periods (Table 1, Figure 1).

In the intragroup analysis (Table 1) between the pre- and postoperative periods, a significant reduction from 0.229 mL to 0.096 mL in the hAM group (*p* = 0.001) and from 0.220 mL to 0.127 mL in the BMSC group (*p* = 0.004) was identified. In the control group, there was a non-significant reduction from 0.182 mL to 0.146 mL (*p* = 0.118).

#### 3.1.3. Left Ventricular End-Diastolic Volume (LVEDV)

The intergroup analysis showed values of 0.294 mL for the control group, 0.330 mL for the hAM group, and 0.312 mL for the BMSC group during the preoperative evaluation (*p* = 0.706). After 30 days, the control group presented values of 0.231 mL, 0.183 mL for the hAM group, and 0.225 mL for the BMSC group (*p* = 0.150).

During the intragroup analysis between the 7th and 30th days, we identified an improvement of LVEDV in both the hAM and BMSC groups (0.330 mL to 0.183 mL, with *p* = 0.002 and, 0.312 mL to 0.225 mL with *p* = 0.011, respectively) (Table 1, Figure 1).

### 3.2. Analysis of the Infarct Area and Collagen

**Figure 2:**
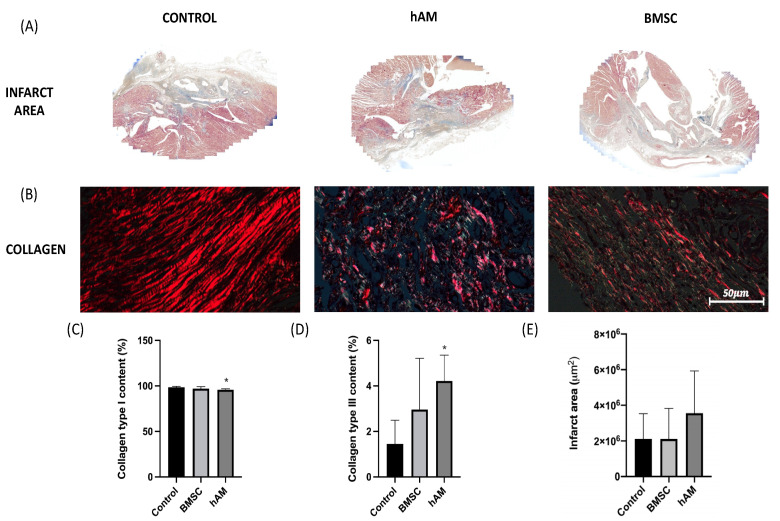
Infarct area and collagen content analysis after 30 days. (**A**) Infarct area quantified as Masson’s Trichrome stain; Images 1×. (**B**) Collagen deposition quantified as Sirius Red positive areas; Images 40×, scale bar = 50 μm. (**C**) Collagen type I content. (**D**) Collagen type III content. (**E**) Infarct area. Results are shown as mean ± standard deviation. *p* < 0.05 denoted statistical significance in comparison to control group (*). *BMSC*: bone marrow mononuclear stem cells; *hAM*: human amniotic membrane.

For the analysis of the infarct area, sections stained with Masson’s Trichrome were analyzed, and the infarct area was denoted according to the percentage of stain in the sample (Figure 2). During histopathological analysis, we observed no difference in the infarct area between the three groups after 30 days of analysis (*p* = 0.383). Type I collagen levels were higher in the control group (*p* = 0.014), while type III collagen levels were higher in the hAM group, with a statistically significant difference in comparison to the control group (*p* = 0.002). The BMSC group showed no significant difference in collagen type I and III levels when compared to each other (Table 3 and Table 4, Figure 2).

### 3.3. Factor VIII

**Figure 3:**
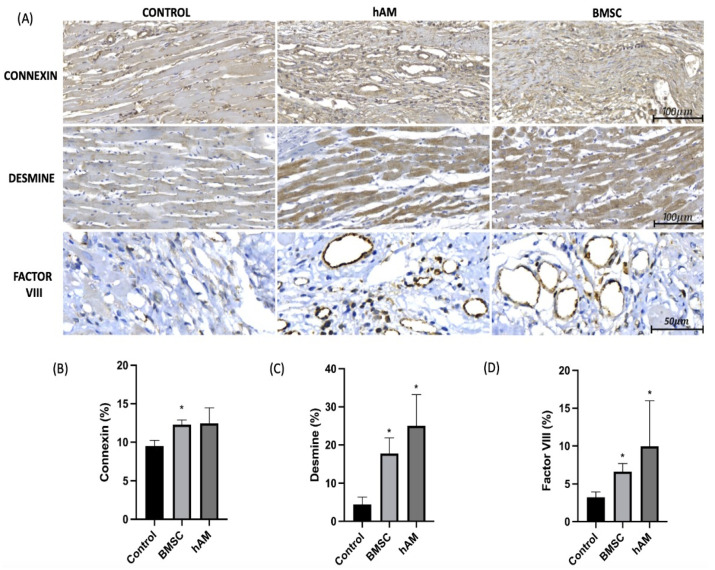
Representative areas of Connexin, Desmine and Factor VIII after 30 days. (**A**) Immunohistochemical representative images for Connexin, Desmine and Factor VIII; Connexin and Desmine images 10×, scale bar = 100 μm; Factor VIII images 20×, scale bar = 50 μm. (**B**) Connexin intergroup analysis after 30 days. (**C**) Desmine intergroup analysis after 30 days. (**D**) Factor VIII intergroup analysis after 30 days; Results are shown as mean ± standard deviation. *p* < 0.05 denoted statistical significance in comparison to control group (*). *BMSC*: bone marrow mononuclear stem cells; *hAM*: human amniotic membrane.

In order to evaluate the angiogenic potential of stem cells and amniotic membrane, the levels of factor VIII in the infarct area were evaluated by immunohistochemistry 30 days after AMI. The results (Table 5, Figure 3) showed a significant increase in factor VIII in both BMSC and hAM groups compared to the control group, but without significant difference when compared to each other (BMSC × hAM) (Table 6).

### 3.4. Desmine

The presence of desmine in the cardiac tissue was evaluated in order to identify cardiac muscle cells in the infarct region. There was a significant increase in desmine in the BMSC and hAM groups compared to the control group (Table 5, Figure 3). Despite this, there was no significant difference when the stem cell and amniotic membrane groups were compared among themselves (Table 6).

### 3.5. Connexin

The levels of Connexin-43 were evaluated in order to identify GAP junctions in the infarct region. The results (Table 5, Figure 3) showed a higher presence of Connexin-43 in the BMSC and hAM groups, with only the BMSC group showing a significant difference in relation to the control group. There was no significant difference when the stem cell and amniotic membrane groups were compared among themselves (Table 6).

## 4. Discussion

The recovery of the contractile function of the heart after an ischemic event that evolved to transmural fibrosis is still a challenge in medical practice. Thus, the evolution to heart failure is inevitable in most cases, mainly due to the extent of the infarct area and the pathological cardiac remodeling [29]. In this scenario, the use of compatible biomaterials with cell-regeneration potential emerges as a therapeutic alternative after AMI [11].

Bone marrow stem cells have been used since 2001 for the treatment of cardiovascular diseases, due to their potential for self-regeneration, proliferation and differentiation into other cell lines [30]. Nevertheless, the mechanisms of interaction and functional consequences after an ischemic event are still controversial. Animal studies [14,22] and clinical trials [31,32] have already demonstrated improvements in cardiac function after bone marrow stem cell therapy following AMI. On the other hand, the results of randomized studies such as the HEBE trial, TIME trial, and REGENT trial have not demonstrated the same potential [33,34,35].

In the present study, we observed a significant benefit in cardiac function with the therapeutic use of stem cells on the 30th day after AMI, with an EF ranging from 30.67% to 46.49% during the follow-up. Moreover, there was significant improvement on ventricular remodeling, with a significant reduction in end-systolic (0.220 mL to 0.127 mL) and end-diastolic (0.312 mL to 0.225 mL) volumes (Figure 1). This anti-remodeling effect has already been observed using a co-culture of mononuclear cells and skeletal myoblasts in a model of Chagas’ cardiomyopathy [36], but it was not observed in a model of transmural myocardial infarction with the same co-culture of cells [37]. This consideration suggests that the mechanisms of action of stem cells may be potentiated when the mononuclear cells are injected into the area of fibrosis and the transition zone between fibrosis and intact myocardium, as used in the present study.

Our results also demonstrated significant benefits in angiogenesis and cellular regeneration in the BMSC group, confirmed with immunohistochemistry by high levels of factor VIII, Connexin, and Desmine, in comparison to the control group (Figure 3). This fact reinforces the hypothesis that the mechanism of action of stem cells involves mainly autocrine and paracrine actions in the cellular microenvironment, and not the direct differentiation into cardiomyocytes as previously considered [11].

The therapeutic efficacy of stem cells on the myocardium depends on their survival after delivery to the myocardium, since these cells need to survive, multiply, and differentiate in the hypoxic microenvironment after AMI [38]. The delivery of the cells can be performed by the venous, intracoronary, venous sinus and intramuscular systems [39]. In our study, we chose the intramuscular method because of its easy and highly efficacy due to the precise delivery of the cells into the ischemic area [40,41].

Human amniotic membrane, in the same way, is also widespread as a regenerative therapy for skin injuries and ophthalmological and gynecological conditions due to its anti-inflammatory and antimicrobial actions [19,20,21], adding to its ability of not generating an inflammatory response in the host tissue when used in a decellularized way [21,22].

In relation to the hAM group, our results showed a recovery in global cardiac function measured through the variation in EF, from 33.21% to 49.77% (*p* < 0.001) after 30 days, associated with a significant reduction in pathological ventricular (Figure 1), similar outcomes to those exposed by Henry et al. [42], Kim et al. [43], and Fang et al. [44] in their studies. These processes occurred without significant modification of the infarct area during histopathological analysis in 30 days, similarly to the study conducted by Roy et al. [16]; the same fact in the infarcted area was observed in the BMSC group (Table 3).

The histopathological analysis also showed that the hAM group had higher levels of type III collagen in comparison to type I collagen. Type III collagen is the immature form of type I collagen, possessing mechanical properties of elasticity that confer a better blood-pumping capacity to the heart [45,46], with previous studies already demonstrating that the increase in the collagen III/I ratio was related to a better cardiac function in patients with previous diastolic dysfunction [47]. Therefore, this increase in type III collagen observed in the hAM group suggests the potential immunomodulatory effect of hAM after AMI; this effect, however, was not observed in the stem cell group (Table 3 and Table 4).

In our study, a higher angiogenesis potential was observed in the area of the amniotic membrane application in the ischemic tissue through elevated levels of Factor VIII. Previous studies have been able to corroborate this finding. For example, Francisco et al. [25] detected an increase in the proliferation of capillaries in site of the implantation of amniotic membrane, and Gorjipour et al. [48], Song et al. [49], and Roy et al. [50] had suggested this property of vascular regeneration of the amniotic membrane through the proliferation of Vascular Endothelial Growth Factor (VEGF) in their analysis. Khorramirouz et al., by detecting CD34^+^ cells in infarcted tissue, corroborated the result of angiogenic property of the amniotic membrane [51], and Danieli et al., evaluating the relationship of the amniotic membrane with endothelial progenitor cells in vitro, detected that it has a strong impact on the formation of new vessels [52].

Moreover, in our study it was possible to evaluate the presence of cardiac regeneration in the hAM group through the quantification of desmine and Connexin in the infarcted tissue, variables with similar outcomes also analyzed by Khorramirouz et al. [51] and Tsuji et al. [53]. Gorjipour et al. demonstrated potential cardiac cell proliferation with the use of amniotic membrane through the presence of cardiac Troponin T [48], and Fang et al., in order to detect the potential for cell differentiation of amniotic membrane, observed the presence of a myosin heavy chain after its application in ischemic tissue [44], corroborating the results of our study. The regeneration of cardiomyocytes and angiogenesis, observed at the site of membrane implantation, allows us to suggest that the therapeutic effects of hAM can be used in the correction of other clinical conditions, such as left ventricle aneurysm, as well as in the correction of structural defects in pediatric heart diseases.

In our study, we chose to use the acellular form of human amniotic membrane, because the absence of the epithelium can contribute to the improvement of biocompatibility of the material, reducing the immunogenic response of the host [11], without losing collagen proteins and growth factors, which are preserved during the decellularization process [21]. Furthermore, the presence of connexin-43 observed in the hAM group demonstrated its ability to incorporate into the myocardium, making use of the amniotic membrane as an attractive biomaterial after AMI, as well as serving as a suitable three-dimensional support matrix for diverse cell populations, stimulating cell immigration and the possible differentiation of neighboring cells [19,20,54].

To our knowledge, this is the first study to compare in the same model the use of bone marrow mononuclear stem cells and human amniotic membrane in the context of heart failure, confirming the therapeutic potential of these agents in the recovery of heart function and reduction in pathological remodeling.

Despite this, limitations such as the animal model and the lack of follow-up over a longer period of time should be pointed out.

## 5. Conclusions

We evaluated the effects of human acellular amniotic membrane and stem cells after myocardial infarction in rats. The results suggest the therapeutic potential of these agents, with improvements in ejection fraction and ventricular anti-remodeling effect, associated with a potential for angiogenesis and cardiomyocyte regeneration in a 30-day follow-up analysis.

## Figures and Tables

**Table 1:** Intragroup analysis of pre- and post-implantation of hAM and BMSC.

Variable	Group	7th Day	30th Day	*p* (7th × 30th Day Echo)
EF (%)	Control (n = 8)	38.73 ± 7.68	39.13 ± 6.54	0.896
	hAM (n = 8)	33.21 ± 7.62	49.77 ± 9.91	0.001
	BMSC (n = 11)	30.67 ± 8.80	46.49 ± 6.68	<0.001
LVESV (mL)	Control (n = 8)	0.182 ± 0.052	0.146 ± 0.056	0.118
	hAM (n = 8)	0.229 ± 0.104	0.096 ± 0.047	0.001
	BMSC (n = 11)	0.220 ± 0.078	0.127 ± 0.058	0.004
LVEDV (mL)	Control (n = 8)	0.294 ± 0.053	0.231 ± 0.073	0.053
	hAM (n = 8)	0.330 ± 0.118	0.183 ± 0.051	0.002
	BMSC (n = 11)	0.312 ± 0.080	0.225 ± 0.069	0.011

*Seventh day*: echocardiographic evaluation before the amniotic membrane and stem cells implantation; *Thirtieth day*: echocardiographic evaluation after 30 days of coronary occlusion; *EF*: ejection fraction; *LVESV*: left ventricular end-systolic volume; *LVEDV*: left ventricular end-diastolic volume; *hAM*: amniotic membrane; *BMSC*: bone marrow mononuclear stem cells. Data are shown as mean ± standard deviation. Values of *p* < 0.05 denote statistical significance.

**Table 2:** Comparison of groups two by two regarding ejection fraction (EF) 30 days after coronary occlusion.

Intergroup Comparison	30th Day EF
	*p*
Control × hAM	0.006
Control × BMSC	0.034
hAM × BMSC	0.326

*Thirtieth day EF*: EF after 30 days of coronary occlusion; *hAM*: amniotic membrane; *BMSC*: bone marrow mononuclear stem cells; Values of *p* < 0.05 denote statistical significance.

**Table 3:** Intergroup analysis of infarct area and collagen types after 30 days after infarct induction.

Variable	Group	Mean	Standard Deviation	*p* (Comparison of 3 Groups)
Infarct area (%)	Control (n = 8)	2,111,209	1,419,393	
	hAM (n = 8)	3,555,715	2,373,125	
	BMSC (n = 11)	2,809,873	1,713,152	0.383
Type I	Control (n = 8)	98.6	1	
Collagen (%)	hAM (n = 8)	95.8	1.1	0.014
	BMSC (n = 11)	97	2.2	
Type III	Control (n = 8)	1.45	1.04	
Collagen (%)	hAM (n = 8)	4.22	1.13	0.014
	BMSC (n = 11)	2.96	2.25	

Values expressed in μm^2^. *hAM*: amniotic membrane; *BMSC*: bone marrow mononuclear stem cells; Values of *p* < 0.05 denote statistical significance.

**Table 4:** Comparison of groups two by two regarding collagen I and III.

Comparison Groups	*p*
Control × BMSC	0.084
Control × hAM	0.002
BMSC × hAM	0.070

*hAM*: amniotic membrane; *BMSC*: bone marrow mononuclear stem cells; Values of *p* < 0.05 denote statistical significance. The *p* values for collagen I and III are the same because collagen I + collagen III = 100%.

**Table 5:** Intergroup analysis of the evaluation of Conexin-43, Desmine and Factor VIII in the infarct area 30 days after infarct induction.

Variable	Group	Mean	Median	Standard Deviation	*p* (Comparison of 3 Groups)
Connexin (%)	Control (n = 4)	9.51	9.39	0.73	
	hAM (n = 4)	12.44	12.04	2.03	0.034
	BMSC (n = 4)	12.27	12.28	0.62	
Desmine (%)	Control (n = 4)	4.41	3.61	1.98	
	hAM (n = 4)	25.01	26.60	8.19	0.015
	BMSC (n = 4)	17.74	18.41	4.12	
Factor VIII (%)	Control (n = 4)	3.23	3.47	0.71	
	hAM (n = 4)	9.95	7.23	6.04	0.018
	BMSC (n = 4)	6.63	6.48	1.04	

*hAM*: amniotic membrane; *BMSC*: bone marrow mononuclear stem cells; Values of *p* < 0.05 denote statistical significance.

**Table 6:** Two by two comparison of the groups regarding Conexin-43, Desmine, and Factor VIII in the infarct area 30 days after infarct induction.

Comparison Groups	*p*		
	Connexin	Desmine	Factor VIII
Control × BMSC	0.018	0.022	0.024
Control × hAM	0.058	0.002	0.003
BMSC × hAM	1.000	0.313	0.622

*hAM*: amniotic membrane; *BMSC*: bone marrow mononuclear stem cells; values of *p* < 0.05 denote statistical significance.

## Data Availability

The data presented in this study are available on request from the corresponding author.
